# Is retirement good for your health? A systematic review of longitudinal studies

**DOI:** 10.1186/1471-2458-13-1180

**Published:** 2013-12-13

**Authors:** Iris van der Heide, Rogier M van Rijn, Suzan JW Robroek, Alex Burdorf, Karin I Proper

**Affiliations:** 1 Centre for Nutrition, Prevention and Health Services, National Institute for Public Health and the Environment, Postbus 1, 3720 BA Bilthoven, Netherlands; 2 Department of Public Health, Erasmus MC, University Medical Center Rotterdam, Rotterdam, Netherlands

**Keywords:** Retirement, Physical health, Mental health, Perceived health, Systematic review, Meta-analysis

## Abstract

**Background:**

Several studies regarding the effect of retirement on physical as well as mental health have been performed, but the results thereof remain inconclusive. The aim of this review is to systematically summarise the literature on the health effects of retirement, describing differences in terms of voluntary, involuntary and regulatory retirement and between blue-collar and white-collar workers.

**Methods:**

A search for longitudinal studies using keywords that referred to the exposure (retirement), outcome (health-related) and study design (longitudinal) was performed using several electronic databases. Articles were then selected for full text analysis and the reference lists of the selected studies were checked for relevant studies. The quality of the studies was rated based on predefined criteria. Data was analysed qualitatively by using a best evidence synthesis. When possible, pooled mean differences and effect sizes were calculated to estimate the effect of retirement on health.

**Results:**

Twenty-two longitudinal studies were included, of which eleven were deemed to be of high quality. Strong evidence was found for retirement having a beneficial effect on mental health, and contradictory evidence was found for retirement having an effect on perceived general health and physical health. Few studies examined the differences between blue- and white-collar workers and between voluntary, involuntary and regulatory retirement with regards to the effect of retirement on health outcomes.

**Conclusions:**

More longitudinal research on the health effects of retirement is needed, including research into potentially influencing factors such as work characteristics and the characteristics of retirement.

## Background

Life expectancy is increasing, and, as a result, individuals will live longer after retirement age than ever before, if retirement ages would remain unchanged. Life expectancy at the common current retirement age of 65 has increased substantially over the years; for example, in the United Kingdom it has increased from 16.2 (1993) to 19.0 (2007) years and in the Netherlands from 16.8 (1993) to 19.0 (2007) [1]. Furthermore, due to decreased birth rates, there are fewer adolescents to compensate for elderly’s exit from the labour force, and as a consequence, the balance between employed and unemployed people will shift. Without intervention, it is thought that the ratio of employed to unemployed will increase from 3:1 in 2004 to 1:1 in 2050 in European Union countries [2], which could greatly impact the healthcare burden. Recent European policies have been developed to raise retirement ages in order to reduce the burden on welfare budgets [3]. In this context there is considerable debate about the timing of retirement and its influence on health [4].

Based on Feldman’s often-cited definition [5], the present study defines retirement as 'the exit from labour force, taken by individuals after middle age, and taken with the intention of reduced psychological commitment to work thereafter’. This definition does not explicitly incorporate the range of retirement forms which include voluntary retirement, involuntary retirement and regulatory retirement. Voluntary retirement can be understood as the relative preference for leisure versus continuing work [6] and seems more likely to occur among those who have economic security after the transition, for instance in the form of savings or pension-like benefits [7]. On the other hand, some workers are forced to retire because of corporate reorganisations or due to health reasons, for example [8]. This type of retirement is often referred to as involuntary retirement and can be expected to cause more stress than voluntary retirement, as workers have less control over the situation. Retirement at a statutory retirement age is referred to as regulatory retirement, but sometimes also labelled as involuntary retirement. An important characteristic of regulatory retirement is that it is country specific, given that statutory retirement ages and socially accepted pension ages differ between countries. With all types of retirement, the national economic situation and the availability of pension-like social benefits can influence the meaning and consequences of retirement [9].

The assumption that retirement may affect physical and psychological well-being is based on the idea that retirement is a major life transition, which results in social-psychological transformation [10]. According to the stressful-life-event approach, which forms the basis of much of the literature on the impact of retirement on health, the stress caused by major life events can have repercussions for an individual’s physical and mental well-being [11]. Factors such as desirability, the degree of control, intrinsic values, predictability and irreversibility can contribute to the stressfulness of a life event [11,12]. These factors can be related to the characteristics of work and retirement; for example, when work is physically or mentally demanding retirement might be perceived as desirable, which may reduce the stressfulness of the transition. In other cases, however, retirement may be perceived as the loss of social contacts and intrinsic values, leading to more stress. Furthermore, involuntary retirement might be perceived as more stressful because of a perceived lack of control, as opposed to voluntary (early) retirement.

Studies have shown contradictory results with regards to the relationship between retirement and health. Early studies suggested that there were no detrimental effects on either physical or psychological health after retirement [12,13]. However, later studies have suggested that retirement does contribute to deterioration in health resulting in an increased burden on the healthcare system [14]. The study of Hult and colleagues showed, adjusting for selection based on health, retirement had no effect on mortality [15]. Westerlund and colleagues showed that retirement had a positive effect on mental health and fatigue, but no effect on chronic conditions [16,17]. Thus, evidence on the impact of retirement on one’s physical as well as mental health is ambiguous. Furthermore, the evidence has not yet been systematically summarised.

The aim of this study is to provide a systematic literature review, which summarises the available evidence on the health effects of retirement and describes differences in health effects between types of retirement (voluntary, involuntary or regulatory) and types of work (blue-collar workers and white-collar workers). Blue-collar workers and white-collar workers differ from each other with respect to their physical and mental workload, which could influence the health effects of retirement. This review is relevant for researchers because it addresses challenges in research, but is also relevant for policy makers since it provides insight into the possible health consequences of retirement that may aggravate or alleviate pressures on the healthcare system.

## Methods

### Search strategy and study selection

For the purpose of the present review, a literature search for peer-reviewed publications was conducted by two authors (RR and SR) in PubMed, Embase and Web of Science up to November 11, 2013. The keywords that were used referred to the exposure (retirement), outcome (health-related) and study design (longitudinal designs) (See Table 1). Only studies published in English were included. Based on the title and abstract, two reviewers (RR and SR) independently selected articles for full text analysis. For final inclusion the articles had to fulfil all of the following criteria: I) the study had to utilise either a prospective or retrospective longitudinal design; II) the study had to involve a non-patient population that did not retire due to health-problems/receive a disability pension; III) the study should report on generic measures of health, such as mental health, perceived general health or physical health before and after retirement. This meant that studies that merely compared retirees with a control group, as was the case in the studies of Bonsang and colleagues [18] and Behncke [19], were excluded from this review. A consensus method was used to resolve disagreements. Finally, the references of all included studies were checked for other possibly relevant articles.

**Table 1:** Terms used for the database search in PubMed, Embase and WoS

	
Exposure	Retirement[MeSH] OR retirement[All Fields] OR pensions[MeSH] OR pensions[All Fields] OR pension[All Fields]
Health outcome	(Health[MeSH:NoExp] OR health[tw] OR “well-being”[All Fields] OR “health status”[MeSH:NoExp] OR “health status”[All Fields] OR “health behaviour”[MeSH] OR “health behaviour”[All Fields] OR “health behaviour”[All Fields] OR “physical health”[All Fields]) OR (“mental health”[MeSH] OR “mental health”[All fields]) OR (hospitalization[MeSH] OR hospitalization[All Fields] OR hospitalisation[All Fields]) OR (“quality of life”[MeSH] OR “quality of life”[All Fields]) OR (“chronic disease”[MeSH] OR “chronic disease”[All Fields] OR “chronic illness”)
Study design	(“Cohort studies”[MeSH] OR “cohort studies”[All Fields] OR “cohort study”[All Fields]) OR (“longitudinal studies”[All Fields] OR “longitudinal study”[All Fields] OR longitudinally[All Fields]) OR (“prospective studies”[All Fields] OR “prospective study”[All Fields]) OR (“follow-up studies”[All Fields] OR “follow-up study”[All Fields] OR follow-up[All Fields]) OR (“retrospective studies”[All Fields] OR “retrospective study”[All Fields])

### Data extraction and quality assessment

One reviewer (IH) extracted the relevant data from the selected publications. The study characteristics extracted were target population (setting, age, sex), sample size, follow-up duration, assessment of retirement, type and measure of health outcomes, and key findings. In the case of uncertainty about the extracted data from the included studies, a second reviewer (RR) was consulted. Pairs of authors (from IH, KP and RR) independently scored the quality of each study according to a standardised set of 14 predefined criteria (Table 2) [20-22]. These criteria distinguished between informativeness (I, n = 4) and validity/precision (V/P, n = 10). Each quality criterion was rated as *positive* (+), *negative* (-), or *unknown* (?) as clarified in Table 2. In the case of an unclear or incomplete description of an item, a question mark was assigned, and the first author of the publication was contacted by e-mail to obtain additional information. Scoring agreement was expressed in a percentage of the total number of items scored (n = 308). Disagreement in scores between reviewers was resolved in a consensus meeting. If, after discussion, an agreement could not be reached, a third author (KP or RR) was consulted in order to reach a final conclusion. The total quality score was assigned by counting the number of items scored positively on the validity/precision criteria (V/P). Studies with a minimum of six points (>50%) were regarded as high quality [20].

**Table 2:** **Criteria list for assessment of the methodological quality of prospective cohort studies **[20-22]

**Criteria**^**a**^		**I, V/P**^**b**^	**% studies meeting the item**
**Study population and participation (baseline)**			
1.	Adequate description of sampling frame, recruitment methods, period of recruitment, and place of recruitment^c^	I	64%
2.	Participation rate at baseline at least 80%, or if the nonresponse was not selective	V/P	23%
3.	Adequate description of baseline study sample for key characteristics^c^	I	32%
**Study attrition**			
4.	Provision of the exact *n* at each follow up measurement	I	68%
5.	Provision of exact information on follow-up duration	I	95%
6.	Response at short-term follow-up was at least 80% of the *n* at baseline and response at long-term follow-up was at least 70% of the *n* at baseline	V/P	50%
7.	Information on not selective nonresponse during follow-up measurement^d^	V/P	18%
**Data collection**			
8.	Adequate measurement of retirement status	V/P	23%
9.	Retirement status was assessed at a time prior to the measurement of the health outcome	V/P	86%
10.	Adequate measurement of the health outcome	V/P	64%
**Data analyses**			
11.	The statistical model used was appropriate and point estimates with measures of variability (CI or SE) have been provided	V/P	59%
12.	The number of cases was at least 10 times the number of the independent variables	V/P	100%
13.	Important confounders were identified and there has been adjusted for	V/P	36%
14.	No selective reporting of results	V/P	91%

^a^Rating of criteria: + = yes; - = no; ? = unclear.

^b^I, criterion on informativeness; V/P, criterion on validity/precision.

^c^Adequate = sufficient information to be able to repeat the study; + is given only if adequate information is given on all characteristics.

^d^+ is given only if nonselective dropout on key characteristics (age, gender, retirement status, health outcomes) is reported in the text or tables.

### Data analysis

The collected data from the included studies was pooled when possible—in cases where there was enough homogeneity and if data was available from three or more studies. Homogeneity was assessed based on the type of health outcome and the type of measure of this health outcome. Pooling was done by calculating the mean differences (SD) based on percentages (percentage before retirement minus percentage after retirement) and by calculating the effect sizes (mean difference/SD). 95% confidence intervals around the mean differences were calculated based on *t-*distributions. For studies on perceived general health, only the prevalence of good general health and poor general health were included in the pooling (the prevalence of average health or an equivalent was not included). Evidence from all included studies was summarised by using a best evidence synthesis, based on results from both high and low quality studies. The best evidence synthesis consists of three levels [20]:

1. Strong evidence: consistent findings in multiple (≥ 2) high-quality studies;

2. Moderate evidence: consistent findings in one high-quality study and at least one low-quality study, or consistent findings in multiple low-quality studies;

3. Insufficient/conflicting evidence: only one study available/inconsistent findings in multiple (≥ 2) studies.

Results of the studies reporting on a particular relationship were considered consistent when for at least 75% of the study results were in the same direction, as defined by p < 0.05.

## Results

### Study characteristics

**Figure 1:**
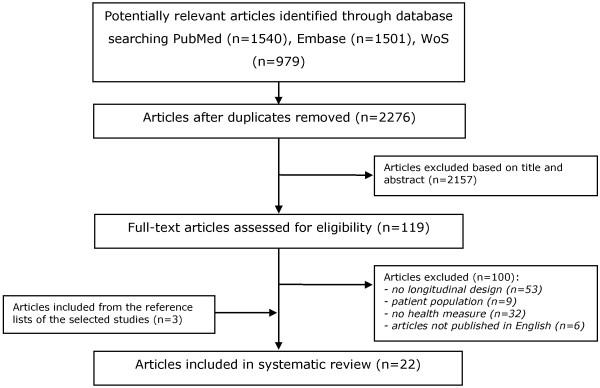
Flow diagram literature search for longitudinal studies on retirement and health.

The literature search resulted in 3182 hits. After removing duplicates (n = 1744), 2276 studies remained, of which 119 were selected based on title and abstract. 19 publications were ultimately included in the review based on the full-text analysis. Three additional studies sourced from the reference lists of the selected publications were also included (see Figure 1). Key characteristics of these studies are presented in Additional file 1, Additional file 2 and Additional file 3. Because of the great diversity of confounders that had already been taken into account by the included studies, it was not feasible for the present study to examine the effect of confounders on the health effects of retirement.

The 22 included studies differed from one other with regards to sample size, measure of retirement, (measure of) health-outcomes, follow-up time, study population and period. Sample sizes ranged from 52 to 14714 (median = 319) [17,23]. For the assessment of types of retirement, three out of the 22 studies used company records or a national database on pensions for statutory age retirement [16,17,24]. The remaining studies used self-reports or did not report on how retirement was assessed. Two studies reported explicitly on early retirement [17,25], ten on old-age (regulatory) retirement [16,17,23-30], two on voluntary retirement [11,26] and one on involuntary retirement [11]. The health outcomes were most often assessed by self-reports (n = 17) and included perceived general health (n = 10) [9,15,21,23,26,28-32]/[11,17,23,28,31-35], mental health (n = 12) [16,23-28,31,36-38] and physical health (n = 12) [16,26-32,34,39-41]. Follow-up times ranged from approximately 1 year to 15 years (see Additional file 1, Additional file 2 and Additional file 3). The included studies stemmed from the United States (n = 7) [29,32,34-39], Israel (n = 2) [25,27], China (n = 1) [30], Northern European countries including Finland (n = 2) [24,28] and Sweden (n = 2) [33,40], and Western European countries including the Netherlands (n = 1) [11], Switzerland (n = 1) [25], France (n = 2) [16,17] and the United Kingdom (n = 4) [26,27,31,41]. As to time-period, eleven of the included studies were published after the year 2000 [11,16,17,23-27,30,37,38], four between 1990–2000 [28,31,33,36], six were published between 1980–1990 [29,32,34,35,39,40], and one was published in 1966 [41].

### Methodological quality assessment

The scoring of the twenty studies led to an agreement of 80%. Ten studies received a question mark for one or more items. Contact information was available for seven authors and six of them replied to requests for additional information. The proportion of the studies meeting the quality criteria varied considerably per criterion, as presented in Table 2. The most common limitation regarding the validity/precision criteria was the lack of information on selective non-response during follow-up, which was met only by 18% of the studies. The most common information criterion not met (32%) was the adequate description of the baseline study sample for key characteristics. Half of the studies were judged as being of high quality (n = 11).

### Perceived general health

**Figure 2:**
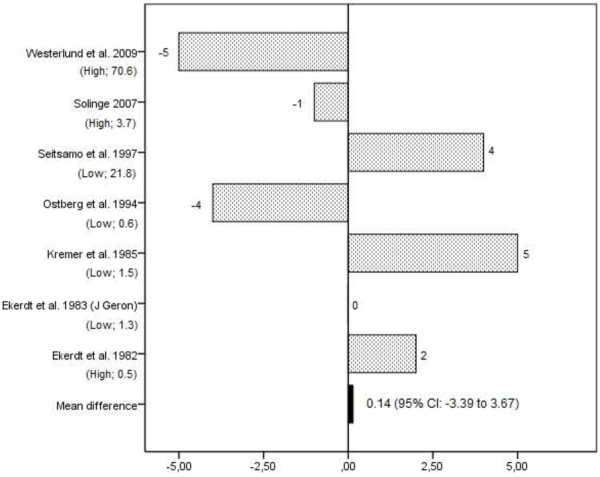
**Difference in the prevalence (%) of poor perceived general health between follow-up (after retirement) and baseline (prior retirement) in longitudinal studies on retirement and health.** The study quality and the relative weight of the study in % are presented in brackets under the studies listed on the Y-axis.

**Figure 3:**
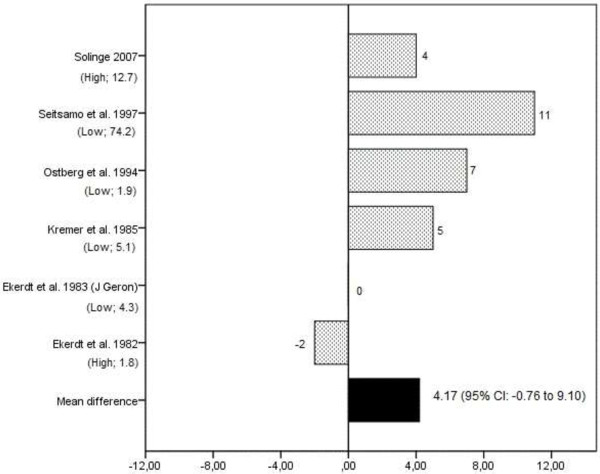
**Difference in the prevalence (%) of good perceived general health between follow-up (after retirement) and baseline (prior retirement) in longitudinal studies on retirement and health.** The study quality and the relative weight of the study in % are presented in brackets under the studies listed on the Y-axis.

Ten studies investigated the effect of retirement on perceived general health (see Table 3 and Additional file 1) [11,17,23,25,28,31-35], of which five were judged as being of high quality [11,17,23,34,35]. Seven studies provided data on the prevalence of poor perceived health before (T1) and after (T2) retirement [11,17,28,32-35]. For these studies, the difference between measurement over time ranged from -5%, indicating a decrease in the prevalence of poor perceived health after retirement, to 5%, indicating an increase in the prevalence of poor perceived health after retirement (see Figure 2). The pooled mean difference was 0.14% (95% CI: -3.39 to 3.67) with an effect-size of 0.04. Six studies provided data on the prevalence of good perceived health before and after retirement [11,28,32-35]. The difference over the follow-up period ranged from -2% to 11% (see Figure 3). The pooled mean difference was 4.17% (95% CI: -0.76 to 9.10) with an effect size of 0.88.

**Table 3:** Evidence for the impact of retirement on various health outcomes from longitudinal studies on retirement and health

**Outcome**	**Impact of retirement**^**a**^		**Best evidence synthesis**^**b**^
	***High quality studies***	***Low quality studies***	
Perceived health	0 ^23^	0 ^25, 31, 32^	Insufficient evidence
	+ ^11, 17^	+ ^28^	
	- ^35^		
	? ^34^	? ^33^	
Mental health	+ ^16, 23, 24, 26, 27, 36^	+ ^25, 31, 37, 38^	Strong evidence
		0 ^28, 30, 31^	
Physical health			
Somatic complaints	0 ^29^		Insufficient evidence
Physical functioning	- ^26, 27^	0 ^38^	Insufficient evidence^c^
Physical fatigue	+ ^16^	0 ^31^	Insufficient evidence
Disability		? ^41^	Insufficient evidence
Chronic or (irreversible) illness	0 ^16^	0 ^40^	Insufficient evidence
	- ^39^	- ^28^	
		+ ^31^	
Perceived serious health problems	0 ^34^		Insufficient evidence

^a^? = unclear (including not shown to be statistically significant); 0 = no change; + = improvement; - = decline.

^b^Strong evidence: consistent findings in multiple (≥ 2) high-quality studies; Moderate evidence: consistent findings in one high-quality study and at least one low-quality study, or consistent findings in multiple low-quality studies; Insufficient evidence: only one study available or inconsistent findings in multiple (≥ 2) studies. Consistency of results was determined following that for at least 75% of the studies reporting on a particular relation the results should be in the same direction, defined by p < 0.05 [20-22].

^c^Insufficient evidence because both studies use data from the same study.

Based on a qualitative assessment using a best evidence synthesis which included all ten studies, conflicting evidence for retirement having an effect on perceived general health was found; the outcomes of the studies varied greatly with some indicating better general health after retirement [11,17,28], one indicating a decrease in general health after retirement [36], and some indicating no effect [21,25,31,32] or an unclear effect (that is not shown to be statistically significant) [33,34] (see Table 3). Only one study distinguished between voluntary and involuntary retirement, showing an improvement in overall perceived general health after retirement [11]. However, those who had retired involuntary were more likely to perceive a decline in their health after retirement than those who had retired voluntarily [11]. The other studies merely reported on regulatory retirement or did not specify retirement type. Three studies specified whether their sample included blue-collar workers, white-collar workers or both, but reported no differences in terms of the beneficial or adverse effects of retirement on perceived health [11,17,35].

### Mental health

Twelve studies reported on the mental health effects of retirement [16,23-28,30,31,36,38,39], of which six were judged as being of high quality [16,23,24,26,27,36] (see Table 3 and Additional file 2). Mental health was operationalised in various ways including well-being and distress [23,30,31], depressive symptoms [16,35,38] and antidepressant use [24]. There was not enough homogeneity between studies regarding the measure of mental health to perform a meta-analysis. Based on a best evidence synthesis, strong evidence was found indicating the beneficial effect of retirement on mental health (see Table 3). Ten studies indicated that retirement is beneficial for various measures of mental health [16,23-27,31,36,38,39], and three studies found no effect [28,30,31]. Most studies presented insufficient information as to the type of retirement and the job characteristics of the sample. One study compared regulatory and voluntary early retirees and found that mental health improved among both type of retirees [26].

### Physical health

**Figure 4:**
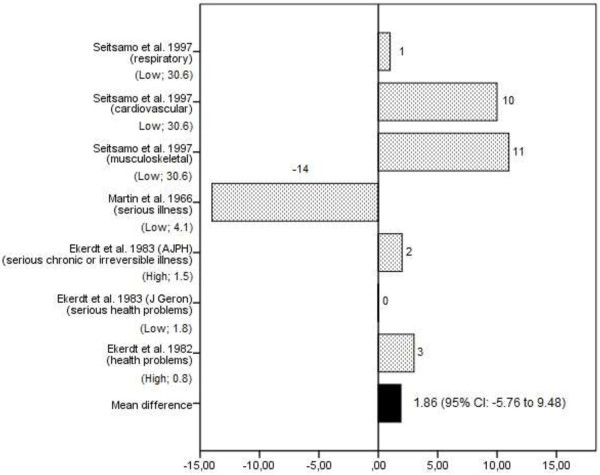
**Difference in the prevalence (%) of poor physical health between follow-up (after retirement) and baseline (prior retirement) in longitudinal studies on retirement and health.** The study quality and the relative weight of the study in % are presented in brackets under the studies listed on the Y-axis.

Twelve studies reported on the physical health effects of retirement [16,26-29,31,32,34,38-41] (see Table 3 and Additional file 3). Six of these were of high quality [16,26,27,29,34,39]. Measures of physical health included somatic complaints [29], physical functioning [26,27,38], physical fatigue [16], having symptoms of an illness [37], the presence of a disability, an (irreversible) illness or a chronic illness [16,28,31,39], and having serious health problems [34]. Six studies provided data on the prevalence of poor physical health before and after retirement [16,28,29,34,38,39]. One study reported three physical health outcomes; these were separately included in the meta-analysis [28]. Differences between follow-up and baseline ranged from -14% to 11%, as presented in Figure 4. The pooled mean difference was 1.9% (95% CI: -5.76 to 9.48) with an effect-size of 0.22. With respect to the eventual change in prevalence of good physical health, not enough data was available.

A qualitative assessment using a best evidence synthesis including all twelve studies, provided conflicting evidence for retirement having an effect of on physical health (see Table 3), since some studies indicated a physical health improvement after retirement [16,31], some indicated a decline [26-28,39], and some indicated no effect [16,29,31,34,38,40] or an unclear effect [41]. Differences between voluntary, involuntary and regulatory retirement were difficult to identify, as most studies did not specify the type of retirement. One study reported that a decline in physical health was found in both voluntary early retirees and regulatory retirees from the civil service [26]. As to differences between blue-collar workers and white-collar workers, studies that drew comparisons between both types of workers found no differences in the effect of retirement on physical health [28,40]. One study examined the prevalence of cardiovascular disease before and after retirement and found a higher prevalence among blue-collar workers as compared to white-collar workers before retirement, but a lower prevalence as compared to white-collar workers after retirement [28].

## Discussion

The present study aimed to systematically assess the literature regarding the effect of retirement on perceived health, physical health and mental health. The results show that the effect of retirement varies between health outcomes. Meta-analyses suggest that retirement has no univocal effect on perceived general health and physical health (i.e. chronic illnesses, serious health problems), since the confidence intervals around the mean difference included both positive and negative values. Best evidence synthesis also indicated conflicting evidence for retirement having an effect on perceived general health and physical health and strong evidence for retirement having a beneficial effect on mental health (i.e. depression, distress and well-being). Few studies looked at the effect of the type of retirement (voluntary, involuntary or regulatory) on health after retirement. One study indicated that involuntary retirees are more likely to perceive a decline in perceived general health after retirement than voluntary retirees [11]. However, other studies indicated no differences between regulatory retirees and voluntary retirees in terms of health after retirement [26,29,39]. Regarding occupational characteristics, this review indicated no clear differences between blue-collar workers and white-collar workers regarding the health effects of retirement [11,17,28,35,40].

The improvement in mental health shortly after retirement may be linked to a reduction in (work related) stress. A study by Westerlund, which involved trajectories of depression seven years before and seven years after retirement, underlined this short term effect [16]. In that study, occupational risk groups showed a steeper decline in depression than non-risk groups, which underlined the fact that work can be a stress-factor. Two studies suggested that improvements in mental health after retirement were merely significant among men [16,27]. The fact that a woman’s primary role used to be in the home, where a man’s primary role used to be at work, might contribute to differences in adjustment after retirement and subsequently to health after retirement [12]. However, the empirical evidence for this reasoning is ambiguous [42].

Two studies indicated retirement having a positive effect on physical health [16,31]. Yet it remains unclear whether these improvements can be explained by factors such as the elimination of adverse work, the reduction of both physical and mental demands, or by positive lifestyle changes. Conversely, deterioration in physical health might also be explained by factors such as the loss of highly valued work, the reduction of physical and mental demands and negative changes in lifestyle [43]. Especially the impact of retirement on the incidence of chronic or severe illnesses needs to be further explored in future research as this may provide further insight into the increased burden on healthcare after retirement due to (chronic) illness.

Few studies examined the effect of the type of retirement on health. A possible reason for the lack of evidence for the differential effects of retirement provisions may be that studies often did not specify the type of retirement. Solinge indicated that it is not necessarily the type of retirement that influences health after retirement, but the extent to which people feel in control at the moment of retirement [11]. Therefore, when workers perceive little or no control regarding this transition, it might lead to stress and subsequently to reduced health [11]. Although studies on retirement due to health reasons were not included in the current review, there may be some ambiguity in the definitions of voluntary and involuntary retirement in the included studies, since it was not always clear from the information presented whether studies could differentiate sufficiently between health-based and other routes into retirement. Some of the included studies reported on retirement for health reasons, in addition to other types of retirement, indicating that those who retire for health reasons seem to benefit from retirement, or benefit more from retirement than those who do not retire for health reasons [16,17,24,26].

Although no clear differences between blue-collar workers and white-collar workers were found regarding the health effects of retirement [11,17,28,35,40] some studies demonstrated associations with certain job characteristics. For example, Westerlund and colleagues showed that a poor work environment and high job demands, both physical and psychological, were associated with greater benefit from retirement in terms of improved self-rated health after retirement [17]. Another study found that a decline in health was only present among those in the highest level of the civil service [27], whereas another study indicated that the prestige of the occupation did not influence the health effects of retirement [29].

A number of sources of inconsistency could explain the differences in the findings of the studies included in this review, including the study quality and follow-up times. Study quality might be a source of inconsistency; since high quality studies are expected to have a lower risk of bias, they might result in other outcomes than those studies with a higher risk of bias. Study quality could have contributed to the inconsistency that was found between the studies reporting on the relationship between retirement and physical health. The studies that found a decline in physical health were most often judged as being of high quality, whereas the studies that found no effect were most often judged as being of low quality. As to perceived general health status and mental health, study quality does not seem to be a source of inconsistency.

A second source of inconsistency might be differences in follow-up times, as it seems most likely to find retirement having an effect on health shortly after retirement. At later follow-up measurements, it becomes increasingly difficult to distinguish between the effect of retirement and other factors that could affect health, such as aging, changes in social contacts and changes in income. In terms of the stressful-life-event approach, feelings of stress or relief and their repercussions on health are expected to occur shortly after the transition into retirement. Concerning our findings, studies with follow-up times of three years or longer were more likely to find improved perceived general health after retirement than studies with follow-up times within three years of retirement [11,17,25,28]. As for mental health, the included studies were comparable regarding follow-up times, which might have contributed to the similarity in study results. Inconsistency in study findings regarding the relationship between retirement and physical health is unlikely to be due to follow-up duration, since the studies were comparable concerning follow-up times, with the exception of one study that reported yearly measures over 15 years [26].

Besides study quality and follow-up duration, other factors such as the country of the study population, the measure of retirement and the age at retirement could also help to explain inconsistencies between study results. Countries differ regarding statutory retirement ages, socially accepted retirement ages and financial benefits after retirement, which affects the number of years people have been in the labour force before retirement and also possibly affects their overall perception of retirement. In the present review, seven studies stemmed from the United States, where there is no statutory retirement age. Twelve stemmed from European countries, where there is a statutory retirement age (which has been set at different ages throughout the years). In order to see whether living in a country with or without a statutory retirement age can explain inconsistency between findings, we compared studies from the United States with the European studies in this review; however, we could not identify a pattern in study findings according to the country in which the study was conducted. This may be due to the limited number of studies included in this review, as well as the variety in health outcomes.

As to the quality assessment criteria, the measure of retirement seems essential, but various studies did not specify how retirement was assessed and when the exact transition into retirement was made between the baseline and follow-up. Lack of information on the exact moment of transition makes it difficult to link changes in health to the transition into retirement. Another issue concerning inconsistency was that in some studies retirement was defined as the complete exit from the labour market, whereas in other studies retirement referred to not being in the full-time labour force or receiving a pension.

Furthermore, with respect to physical as well as mental health outcomes, mostly self-reports were used. Although self-reports provide important insights, in order to get a more complete picture of the relationship between retirement and health, the use of more objective measures, such as medicine prescriptions, as was done in the study of Oksanen and colleagues [24], is desirable. It seems that the greatest lack of evidence exists regarding the effect of retirement on diagnosed health conditions.

An important point regarding the interpretation of the evidence is the fact that various studies stated that it is complex to distinguish between the effect of retirement and the effect of aging. To control for the effect of aging, some studies combined the longitudinal design with a cross-sectional design by including a control group who remained in the labour force. For instance, this design was adopted by Mein and colleagues, who found little difference in physical function between those who retired and those who remained in labour force [27]. On the other hand, findings from the same study indicated that mental health declined among those who continued to work and improved among those who had retired [25]. Another study suggests that mental health is better after voluntary early retirement and statutory retirement as compared to being in the workforce [26]. These findings suggest that changes in health after retirement could be attributed to the act of retiring itself, rather than the process of aging. However, more research is needed to provide evidence that the effect of retirement is truly distinct from the effects of aging, preferably by the use of interrupted time-series in accordance with the studies of Westerlund and colleagues [16,17]. Following this, it might be valuable to explore to what extent age at retirement affects health, since it has been suggested that age at retirement is an important predictor of health after retirement [44].

Since most studies in this review were conducted after the year 2000, the health effects of retirement seem to be of current interest. Furthermore, strong evidence of the positive effect of retirement on mental health indicates that there may be other extenuating factors at work associated with reduced mental health among older workers. Therefore, attention to the prevention of reduced mental health in the years before retirement is needed.

For the purpose of the present study only retirement was included as pathway into post-working life. However, we acknowledge that there are other pathways into post-working life as well, that do not start with retirement; for example, older workers who are made redundant may decide to retire because of the difficulties these workers face with finding a new job. As to the methods used to assess the evidence, a strength of the present review is the use of a combination of quantitative and qualitative methods. A limitation of the quantitative approach is that fewer studies were available for the meta-analysis as compared to the qualitative approach. Since the studies that reported on mental health were too heterogeneous, we refrained from performing a meta-analysis regarding this outcome. A weakness of the qualitative approach is the subjectivity of the quality rating of the studies, which might therefore be subject to bias.

## Conclusions

This review indicates that retirement can have both beneficial as well as adverse health effects. Strong evidence was found for retirement having a beneficial effect on mental health; conflicting evidence was found for retirement having an effect on perceived general health and physical health. Furthermore, it is important to perform longitudinal studies on the relationship between retirement and health with a sufficient follow-up period, addressing the differences between voluntary, involuntary and regulatory retirement, as well as the differences between blue- and white-collar workers.

## Competing interests

The authors declare that they have no competing interests.

## Authors’ contributions

All authors made significant contributions to the conception of this study. RR and SR conducted the literature search and selected the studies. IH, RR and KP scored the quality of the studies. IH extracted the data and drafted the manuscript with contributions of RR, SR, AB and KP. All authors read and approved the final manuscript.

## Pre-publication history

The pre-publication history for this paper can be accessed here:

http://www.biomedcentral.com/1471-2458/13/1180/prepub

## Supplementary Material

Additional file 1 Longitudinal studies reporting on the relation between retirement and perceived general health. Click here for file

Additional file 2 Longitudinal studies reporting on the relation between retirement and mental health. Click here for file

Additional file 3 Longitudinal studies reporting on the relation between retirement and physical health. Click here for file
