# Supplementary material for: Is retirement good for your health? A systematic review of longitudinal studies
Source: BMC Public Health. 2013 Dec 13;13:1180. doi: 10.1186/1471-2458-13-1180 (PMC4029767; doi:10.1186/1471-2458-13-1180)
Supplement: Additional file 1 — Longitudinal studies reporting on the relation between retirement and perceived general health. [file 1471-2458-13-1180-S1.docx]

| **Additional file 1. Longitudinal studies reporting on the relation between retirement and perceived general health** | | | | | | | |
| --- | --- | --- | --- | --- | --- | --- | --- |
| **Author, year, country** | **Quality** | **Study population** | **Follow up time** | **Assessment of retirement** | **Assessment of**  **health outcome** | **Statistical analysis** | **Results** |
| Erkerdt et al., 1982 [34]  United States | High | Men from the Veterans Administration Normative Aging Study aged 56 to 67 at baseline  n = 559 at baseline (1975)  n = 112 included | 1 year | Self-reported retirement | Self-report perceived health on a 4-point scale | Percentages | Having good perceived physical health dropped from 86.5% to 83.8% and having poor perceived physical health increased from 13.5% to 16.2%. |
| Erkerdt et al., 1983 [32]  United States | Low | Men from the Veterans Administration Normative Aging Study  n = 332 at baseline (1975 or 1978)  n = 263 included | 3 years | Self-report of retirement with no intention to return to full-time employment | Self-report perceived health on a 4-point scale | Percentages | No change was found in perceived health (poor: 4% vs. 4%; fair: 28 vs. 27; good: 118 vs. 120; excellent 50 vs. 49). |
| Gall et al., 1997 [31]  United Kingdom | Low | Male residents of London aged 54 to 69 at baseline  n = 224 at baseline (year not shown)  n = 117 included | T1: 2-4 years prior retirement  T2: 1 year post-retirement  T3: 6-7 years post-retirement | Assessment of retirement not shown | Single item for perceived health (scale not shown)  RDI Health for satisfaction with health | MANOVA | No significant changes were found for perceived health and satisfaction with health when comparing T1 and T2. Satisfaction with health decreased at 6-7 years postretirement (T3) (F(l,113) = 14.50, *p <* .0001). |
|  |  |  |  |  |  |  |  |
| Kremer et al., 1985 [35]  Israël | Low | Male blue- and white-collar workers of three industrial plans in Israel aged 66 to 70 at baseline  n = 400 at baseline (1973 to 1975)  n = 310 included  (retrospective study) | Retrospective: 1-5 years before retirement  T1:6 months to 3.5 years after retirement | Assessment of retirement not shown | Self-report perceived health on a 5-point scale | T-test | A higher value of health state in the period before retirement compared to the period after retirement (M = 3.45 versus M = 2.98, t = 6.7, p < 0.001). |
| Mojon-Azzi et al., 2007 [25]  Switzerland | Low | Individuals aged 55 to 75 years at baseline  n = 2 461 at baseline (1999)  n = 83 included  (47 men; 36 women) | 4 years | Self-reported retirement due to old age or early retirement | Self-reported change in health over 12 months (from 0 = greatly improved  to 10 = greatly worsened) | Changes in the six  health measures for  retired and working  persons as % of men/women whose health improved, remained unchanged, or worsened between  baseline and follow-up | Men: 15% improved health; 75% unchanged health; 10% worsened health.  Women: 25% improved health; 64% unchanged health; 11% worsened health. |
| Nuttman-Schwartz et al., 2004 [23]  Israel | High | Jewish men from 14 workplaces evenly divided between service and production sectors aged 64 at baseline    n = 56 (year not shown)  n = 52 included | T1: 6 months prior retirement  T2: 1 year after retirement | Self-reported retirement | Perceived health status by the Multi-Dimensional Health scale plus one extra item | Analysis of variance | No significant change was found in perceived health prior retirement (M=50.23, SD=7.24) compared to perceived health after retirement (M=49.92, SD=8.47): F(1,48)=0.08. |
| Ostberg et al., 1994 [33]  Sweden | Low | Female municipal employees in Malmö aged 62 to 64 at baseline  n = 116 at baseline (year not shown)  n = 116 included | T1: 6 months prior retirement  T2: 5 months after retirement | Assessment of retirement not shown | Self-report perceived health on a four-point scale | Percentages before and after retirement | Subjective health improved in 25 (22%) and got worse in 10 (9%). |
| Seitsamo et al., 1997 [28]  Finland | Low | Municipal workers aged 55 to 69 at baseline    n = original baseline sample size not shown (1981)  n = 4 534 included  (1877 men; 2657 women) | T1: 1981  T2: 1992  (time before retirement and time after retirement not shown) | Assessment of retirement not shown | Perceived health by subjective assessment of health as compared to others; the extent to which diseases hamper everyday life | Chi-square test | Perceived good health increased after retirement (8% vs. 19%, p<0.001), perceived average and decreased health dropped after retirement (respectively 27% vs. 25% and 50% vs. 37%) and poor health increased (15% vs. 19%). |
| van Solinge, 2007 [11]  The Netherlands | High | Employees aged 55 or older at baseline working in 50+ companies of retail and trade and industry. (Panel study on retirement behaviour)  n = 1 058 at baseline (1995)  n = 778 included  (451 men; 327 women) | 6 years | Self-reported voluntary and involuntary retirement (health reasons, organisational reasons or other reasons) | Perceived health by self-assessment of physical health on  a 5-point scale | Chi-square test | Perceived health was more positive following retirement (χ2 = 6.44, *df* = 2, *p* < .05). |
| Westerlund et al., 2009 [17]  France | High | Employees aged 35 to 50 at baseline of the French national gas and electricity company: Electricité de France-Gaz de France (GAZAL cohort)  n = 20 624 at baseline (1989)  n = 14 714 included  (11 581 men; 3133 women) | T1: 1 to 7 years prior retirement  T2: 1 to 7 years after retirement | Date of retirement from company records (statutory age of retirement between 55 and 60 and early retirement based on health grounds) | Perceived health status by self-report on 8-point scale | Percentages before and after retirement  Odds ratio | Suboptimum health fell from 19.2% (95% CI 18.5 – 19.9) to 14.3% (13.7 – 14.9) for both men and women and across occupational grades.  The odds of suboptimum health immediately before and after retirement were higher in those who reported high and intermediate psychological and physical demands compared to low demands. |

Abbreviations: OR = odds ratio; CI = 95% confidence interval; T1 = baseline; T2 = follow-up
