# Supplementary material for: Is retirement good for your health? A systematic review of longitudinal studies
Source: BMC Public Health. 2013 Dec 13;13:1180. doi: 10.1186/1471-2458-13-1180 (PMC4029767; doi:10.1186/1471-2458-13-1180)
Supplement: Additional file 2 — Longitudinal studies reporting on the relation between retirement and mental health. [file 1471-2458-13-1180-S2.docx]

| **Additional file 2. Longitudinal studies reporting on the relation between retirement and mental health** | | | | | | | |
| --- | --- | --- | --- | --- | --- | --- | --- |
| **Author, year, country** | **Quality** | **Study population** | **Follow up time** | **Assessment of retirement** | **Assessment of health outcome** | **Statistical analysis** | **Results** |
| Gall et al., 1997 [31]  United Kingdom | Low | Male residents of London aged 54 to 69 at baseline  n = 224 at baseline (year not shown)  n = 117 included | 14 months to 7 years and 4 months | Assessment of retirement not shown | Psychological distress SCL-90 | MANOVA | Psychological distress decreased 1 year post retirement (F(1,113)=14.64, p<.0001). Psychological distress increased at 6-7 years post retirement. |
| Gayman et al., 2013  [38]  United States |  | Americans aged 51 to 61 at baseline (HRS)  n = 12 654 at baseline (1992)  n = 3 264 included (number of men and women not shown) | Maximum 14 years | Self-reported retirement (partially or fully) | Depressive symptoms by 8 items from the CESD scale | Chi-square test | Whites experienced a significant decrease in depression symptoms (T1 Mean: 0.06 versus T2 Mean: 0.04  (χ2 diff. = 5.49 (df = 1), p = .02)) after retirement, but blacks did not (T1 Mean: 0.08 versus T2 Mean:0.08 (χ2 diff. = 0.01 (df = 1), p = .92)). |
| Jokela et al., 2010 [26]  United Kingdom | High | Civil servants aged 54 to 76 at baseline (Whitehall II)  n = 10 308 at baseline (1985)  n = 7 584 included  (5248 men; 2336 women) | Maximum 15 years | Self-reported as being voluntary early retired, retired or statutory retired (at age 60) | Mental health by the Short Form Medical Outcomes | T-test | Based on figure 2:  Mental health improved with statutory retirement. Early retirement shows first a decline in mental health, than an increase and then a decline again. |
| Mandal et al., 2008 [37]  United States | Low | U.S. residents over the age of 50 at baseline  n = 26 728 at baseline (1992)  n = 3 578 included  (men vs. women not shown) | 10 years | Self-reported retirement (voluntary job loss) | Mental health by 8 items from the CESD scale | (Logistic) regression analyses | Mental health conditions improved after retirement (Est. -0.261 (SE 0.034) p = 0.01.) |
| Mein et al., 2003 [27]  United Kingdom | High | Civil servants aged 54 to 59 at baseline (Whitehall II)  n = 10 308 at baseline (phase one 1985-1988)  n = 392 included  (264 men; 128 women) | The mean interval 3 years (range 23-59 months) | Self-reported retirement  (only those where included who retired at the mandatory age of 60) | Mental health by the short Form 36 General Health Survey | Crude mean change | Mental functioning improved for men (crude mean change 1.56, CI 0.80 to 2.32), but not for women (cured mean change 1.06, CI-0.28 to 2.40). Improvements in mental health functioning among all retirees were observed only among the highest two civil service grades (respectively crude mean change 2.10, CI 1.23 to 2.97; 1.52, CI 0.42 to 2.62). |
| Mojon-Azzi et al., 2007 [25]  Switzerland | Low | Individuals aged 55 to 75 years at baseline  n = 2 461 at baseline (1999)  n = 77 included  (43 men; 34 women) | 4 years | Self-reported retirement due to old age or early retirement | Depression by one item on a 10-point scale | Changes in depression as % of men and women whose health improved (+), remained unchanged (0), or worsened (-) between baseline and follow-up | Men: 40% improved mental health; 35% unchanged mental health; 25% worsened mental health.  Women: 35% improved mental health; 41% unchanged mental health; 24% worsened mental health. |
| Nuttman-Schwartz et al., 2004 [23]  Israel | High | Jewish men from 14 workplaces evenly divided between service and production sectors aged 64 at baseline    n = 56 (year not shown)  n = 52 included | T1: 6 months prior retirement  T2: 1 year after retirement | Self-reported retirement | Mental health (well-being and distress) by the Mental Health Inventory | Analysis of variance | A significant decline in distress was found (M=45.19, SD=19.32 vs. M=39.75, SD=17.05, F(1,48)=4.24).  No significant change in well-being was found (M=61.88, SD=15.36 vs. M=64.18, SD=15.99, F(1,48)=1.12). |
| Oksanen et al., 2011 [24]  Finland | High | Participants from the Finish Public Sector cohort  n = 151 618 at baseline (1994)  n = 11 019 included  (2723 men; 8296 women) | 9 years | Data from the Finnish Centre for Pensions on statutory retirement age. | Mental health by antidepressant use before and after retirement. Data were obtained from the Drug Prescription Register by the Social Insurance Institution of Finland | Prevalence differences and prevalence ratios | There was a decline in antidepressant use from 1 year before to 1 year after the transition to retirement (prevalence ratio: 0.77 CI = 0.68 to 0.88). |
| Reitzes et al., 1996 [36]  United States | High | Men and women aged 58 to 64 at baseline from North Carolina (Raleigh-Durham-Chapel Hill)  n = 826 at baseline (1992)  n = 299 included  (men vs. women not shown) | 2 years | Assessment of retirement not shown | Depression by the CESD scale (self-report) | T-test and Chi-square test | No change was found for self-esteem.  A decrease in depression was found (M =6.2, SD = 6.7 at T1 versus M = 4.7, SD = 5.6 at T2 (p < .01)). |
|  |  |  |  |  |  |  |  |
| Seitsamo et al., 1997 [28]  Finland | Low | Municipal workers aged 55 to 69 at baseline in 1992  n = original baseline sample size not shown (1981)  n = 4 534 included  (1877 men; 2657 women) | T1: 1981  T2: 1992  (time before retirement and time after retirement not shown) | Assessment of retirement not shown | Mental disease by self-report of presence of disease, presence of impairment or injury, diagnose of physician | Pearson’s chi square test and Logistic regression models | No difference was found for the prevalence of mental disorder. |
| Westerlund et al., 2010 [16]  France | High | Employees aged 35 to 50 at baseline of the French national gas and electricity company: Electricité de France-Gaz de France (GAZAL cohort)  n = 20 624 at baseline (1989)  n = 14 104 included  (11 246 men; 2858 women) | 15 years | Date of retirement from company records | Mental fatigue by one item on 8-point scale  Depressive symptoms by the CES-D scale | Logistic regression analyses | Adjusted for time of data collection, retirement was associated with a decrease in the prevalence of mental fatigue (OR: 0.19, CI:0.18 to 0.21) and depressive symptoms (OR: 0.60, CI:0.53 to 0.67). |
| Yeung et al., 2013  [30]  China |  | Workers from public and private organisations in China  n = 127 at baseline  n = 90 included (66 men; 24 women) | 1 year | Assessment of retirement not shown  (Employees who were under mandatory retirement scheme and expected to retire in the next six months were recruited) | Psychological distress by the validated Chinese version of the 12-item General Health Questionnaire.  Psychological well-being by the validated Chinese version of the PWB. | T-test | PWB (T1: M=3.60, SD:0.44; T2: M=3.60, SD:0.44); t: -0.02), and psychological  distress (T1: M=1.75, SD:0.36; T2: M=1.75, SD: 0.36); t: 0.05) remained largely similar over time. |

Abbreviations: OR = odds ratio; CI = 95% confidence interval; T1 = baseline; T2 = follow-up
