# Supplementary material for: Is retirement good for your health? A systematic review of longitudinal studies
Source: BMC Public Health. 2013 Dec 13;13:1180. doi: 10.1186/1471-2458-13-1180 (PMC4029767; doi:10.1186/1471-2458-13-1180)
Supplement: Additional file 3 — Longitudinal studies reporting on the relation between retirement and physical health. [file 1471-2458-13-1180-S3.docx]

| **Additional file 3. Longitudinal studies reporting on the relation between retirement and physical health** | | | | | | | |
| --- | --- | --- | --- | --- | --- | --- | --- |
| **Author, year, country** | **Quality** | **Study population** | **Follow up time** | **Assessment of retirement** | **Assessment of health outcome** | **Statistical analysis** | **Results** |
| Berg et al., 1988 [40]  Sweden | Low | Male workers at the shipyards in Gothenburg  n = 352 at baseline (1982)  n = 327 included | 3 years  T1: 1982  T2: 1985 | Assessment of retirement not shown | Musculoskeletal symptoms by self-reported complaints on neck, shoulder, knees, back and elbows on a 3-point scale | Chi-square test | No statistically significant differences were found for musculoskeletal symptoms before and after retirement for both manual workers and office workers. |
| Erkerdt et al., 1982 [34]  United States | High | Men from the Veterans Administration Normative Aging Study aged 56 to 67 at baseline  n = 559 at baseline (1975)  n = 112 included | 3 years  T1: 1975  T2: 1978 | Self-reported retirement | Self-reported health problems on a 2-point scale | Percentages | Having no health problems increased from 76.6% to 77.5% and having health problems increased from 13.5% to 17.1%. |
| Erkerdt et al., 1983 [39]  United States | High | Men from the Veterans Administration Normative Aging Study aged 49 to 67 at baseline  n = 343 at baseline (year not shown)  n = 229 included | Mean of 3.5 years | Self-reported retirement | Physical health by ratings based on medical examinations, including medical history, physical examination by an internist, ECG, chest x-ray, and standard blood and urine test. Physical health was rated on a 4-point scale | Percentages | Irreversible illness with serious disability increased  (1.8% at T1 vs. 3.5% at T2); serious chronic illness without disability increased  (10.0 vs. 22.7); little change in minor chronic problems (59.8 vs. 58.5); excellent health decreased  (28.4 vs. 15.3). |
|  |  |  |  |  |  |  |  |
| Ekerdt et al., 1983 [29]  United States | High | Men from the Veterans Administration Normative Aging Study aged 49 to 67 at baseline  n = 270 at baseline  n = 171 included | 3 to 5.5 years | Self-report of retirement at compulsory age and claim to not have returned to full-time employment | Somatic complaints by the Cornell Medical Index | Mean increase by Wilcoxon signed-rank test.  Regression analysis | No significant increase in somatic complaints was found after retirement. No significant main effect was found for time spent in retirement, age at retirement, part-time work in retirement, former occupation level, compulsory retirement and marital status. |
| Ekerdt et al., 1983 [32]  United States | Low | Men from the Veterans Administration Normative Aging Study  n = 332 at baseline (1975 or 1978)  n = 263 included | 3 years  T1: 1975 or 1978  T2: 1978 or 1981 | Self-report of retirement with no intention to return to full-time employment | Report of serious health problems coded as no/uncertain/yes | Chi-square test | No significant improved or diminished health after retirement. |
| Gall et al., 1997 [31]  United Kingdom | Low | Male residents of London aged 54 to 69 at baseline  n = 224 at baseline (year not shown)  n = 117 included | T1: 2 to 4 months preretirement  T2: 1 year postretirement  T3: 6-7 years postretirement | Assessment of retirement not shown | Physical illness by the Kaiser Illness Index  Disability by Items and scale not shown | MANOVA | No significant changes were found for disability.  Retirees level of physical illness remained stable to 1 year post retirement, but increased 6-7 years postretirement (F (1,116)=8.34, p<.005). |
| Gayman et al., 2013  [38]  United States |  | Americans aged 51 to 61 at baseline (HRS)  n = 12 654 at baseline (1992)  n = 3 264 included (number of men and women not shown) | Maximum 14 years | Self-reported retirement (partially or fully) | Physical limitations by a shortened version of the ADL/IADL scales. | Chi-square test | Neither Whites  (χ2 diff. = 0.23[*df* = 1], *p* = .63) nor Blacks (χ2 diff. = 3.12 [*df* = 1], *p* = .08)  reported significant changes in physical limitations during the retirement transition. |
| Jokela et al., 2010 [26]  United Kingdom | High | Civil servants aged 54 to 76 at baseline (Whitehall II)  N = 10 308 at baseline (1985)  n = 7 584 included  (5248 men; 2336 women) | Maximum 15 years | Self-report as being voluntary early retired or statutory retired (at age 60) | Physical functioning by the Short Form Medical Outcomes Survey 36 questionnaire | T-scores before and after retirement | Physical functioning declined for both statutory and voluntary early retirees. |
|  |  |  |  |  |  |  |  |
| Mein et al., 2003 [27]  United Kingdom | High | Civil servants aged 54 to 59 at baseline (Whitehall II)  n = 10 308 at baseline (phase one 1985-1988)  n = 392 included  (264 men; 128 women) | The mean interval 3 years (range 23–59 months)  T1: 1991-93 T2: 1995 | Self-reported retirement  (only those where included who retired at the mandatory age of 60) | Physical functioning by the Short Form 36 General Health Survey | Crude mean change | Physical functioning declined among men (crude mean change -1.60, CI -2.42 to -0.78), but not among women (crude mean change -0.91, -2.22 to 0.39). Decline in physical functioning among all retirees was observed only among the highest civil service grade (crude mean change -1.89, CI -2.95 to -0.84). |
| Martin et al., 1966 [41]  United Kingdom | Low | Male employees of Pilkington Bros. Ltd. and National Health patients of the GP’s in town.  n = 604  (retrospective study) | Unclear | Self-reported retirement after the age of 55 | Self-report of being under treatment for a serious illness | Percentages | A drop of nearly 20% in the incidence of serious illness in the two years following retirement was found. |
| Seitsamo et al., 1997 [28]  Finland | Low | Municipal workers aged 55 to 69 in 1992  n = 4 534 at baseline (1981: original sample size not shown)  n = 4 534 included  (1877 men; 2657 women) | T1: 1981  T2: 1992  (time before retirement and time after retirement not shown) | Old age retirement  Assessment of retirement not shown | Cardiovascular disease, musculoskeletal disease, respiratory disease and mental disease based on self-report of presence of disease, presence of impairment or injury, diagnose of physician | Chi-square test | The prevalence of musculoskeletal disease (34% vs. 44%) and cardiovascular disease (17% vs. 27%) increased. No difference was found for respiratory disease and mental disorder. |
| Westerlund et al., 2010 [16]  France | High | Employees aged 35 to 50 at baseline of the French national gas and electricity company: Electricité de France-Gaz de France (GAZAL cohort)  n = 20 624 at baseline (1989)  n = 14 104 included  (11 246 men; 2858 women) | Yearly observations during 7 years before and 7 years after retirement | Receipt of official retirement pension (statutory retirement)  Date of retirement from company records | Coronary heart disease, stroke, diabetes (not specified) and respiratory disease based on self-reports and validated against medically certified sickness absence records.  Physical fatigue based on one item on 8-point scale | Prevalence  Logistic regression analyses | Adjusted for time of data collection, retirement did not change the risk for major chronic diseases. Retirement was associated with a decrease in the prevalence of physical fatigue one year after retirement versus one year before retirement (OR: 0.27, 0.26 to 0.30). |

Abbreviations: OR = odds ratio; CI = 95% confidence interval; T1 = baseline; T2 = follow-up
